# An Address at the Commencement of the Dental College of Ohio

**Published:** 1860-04

**Authors:** B. P. Aydelott

**Affiliations:** President of the Board of Trustees


### AN ADDRESS AT THE COMMENCEMENT OF THE DENTAL COLLEGE OF OHIO, 1860.

BY B. P. AYDELOTT, M. D., D. D.

President of the Board of Trustees.

Every pursuit in life demands more or less study. Some persons indeed seem naturally averse to books; and what little knowledge they acquire by these is a task to which they must be driven. Still, even such persons when they become engaged in the business walks of life, find that they must study these—and that just the more closely too for having neglected their book education; or they will remain mere drudges all their days, ill-informed, discontent, poor, and of little or no influence. To be even a thriving mechanic, or a prosperous man of trade demands no little study of some kind. Whatever our position or calling, the *understanding* as well as the body must be employed, or we shall never accomplish much.

But there are pursuits in life for which early preparatory education is almost indispensable. The few who have eminently succeeded in these without this previous study—such a preparation, we mean, as only books and judicious teachers could give—have triumphed only by laboring the harder to make up for the want of early advantages, or their neglect to improve these. But it is a melancholy fact, that these self-making men frequently sink into a premature grave, broken down by the toils which their circumstances and their aspirations make necessary. Early study would have saved them for usefulness and honor.

And are we asked—what are those pursuits which imperatively demand study at every step, and for which early education too is so indispensable? We answer—the *professions*, technically so called,—more especially those of law, medicine, and theology.

And why is study so important here? Because so many sciences are involved in the knowledge of them that it requires years of toil over books before one can be properly qualified even for a pupilage in the professions; and then other years of similar toil before we are duly prepared for the practice of the profession; and afterward, a whole life of study in order to stand up as masters of the profession; and because also the social position of the man who is engaged in any of the professions, is such as calls for high and varied intelligence and thorough intellectual training, as well as elevated morals and refined manners. Without these qualifications, acquired some how and at some time—and generally the earlier the better—no one can honestly expect or acquire the position which a professional man ought to occupy.

We need not stop to show the truth and the importance of these introductory remarks. No one who has an adequate idea of a profession, and who is sensible of the obligations it imposes, will deny their truth or importance.

We proceed then to bring before you, as briefly as we can consistently with clearness,—the *Subjects*, the *Method*, and the *End* of study. And first of

I. The *Subjects of Study*. That course of study which is adapted to man as man, and to the particular position of the individual is the proper course for him. But man is an intellectual and moral being, and a being susceptible also of the emotions of the sublime and beautiful; and these various capacities, intellectual, moral, and æsthetic, are all associated with a physical structure. In other words,—he has understanding, conscience, and taste embodied in a material frame.

Again:—every department of nature, the whole storehouse of God's works, is capable of shedding light upon the path of the professional man. Every kind of knowledge he may turn to good account; much knowledge is absolutely necessary to him. He ought therefore to be a universal scholar.

We do not mean, indeed, that the professional man ought to be a thorough embodiment of all learning—a sort of walking encyclopedia, useful chiefly for occasional reference by others. Not at all. Such a man—were such an one possible—would utterly fail of professional usefulness and success. We say were such an one possible—because the subjects of knowledge are so numerous and extensive, that no one can be profoundly learned in more than a single department, and often only in a portion of that department.

This, however, we do mean:—he who is preparing for the study of a profession ought to go over the whole outline of science and literature, and thus make himself acquainted with, at least, the general principles of every species of knowledge. It is only then he is best qualified to cultivate with special assiduity those branches of learning involved in the daily and intelligent practice of his profession. In one word,—he should be a good general scholar, and then specially learned in his profession. He who brings such a preparation to his duties can scarcely fail to be useful, successful, honored.

In this day of general education and consequent universal diffusion of knowledge, such are the views commonly entertained concerning the professions. The public expect the professional man not only to be thoroughly learned and skillful in his particular pursuits, but to be a good general scholar also, able to converse well upon scientific and literary subjects, and to be a man of unexceptionable morals, and of gentlemanly culture. It is too late in the day for an ignoramus, or a corrupt man, or a clown to get along in the professions. By force of peculiar circumstances, or by dint of impudence, he may, in certain situations and for a time, succeed, but he will, sooner or later, be found out and despised.

And we ought to be thankful that it is so; it shows progress —healthy progress in the world.

Is it here asked—where may such a preparatory education be best acquired? We answer, without hesitation, in our established colleges. Their course of study, scientific, literary, moral and æsthetic, together with their customary surrounding influences, is well calculated to develop and cultivate the whole man. The youth who goes to a good college determined to make the best use of his opportunities, will be sure to find there competent, and often, very able scientific and literary instructors—men whose example and manners also, taken in connection with the society that usually grows up in the neighborhood of such an institution,—instructors, we say, whose example and manners thus accompanied, can not fail to render him a scholar, a man of principle, and a gentleman. And the youth who can live for years in the midst of such privileges, and yet come out a block-head, a knave, a debauchee, or a clown, shows—not the unfitness of the course of training, but his own unfitness for it. It was a sad mistake to send that young man to college. The ancients had a shrewd saying upon this subject:—Exquovis ligno non fit Mercurius:—a Mercury is not to be carved out of any stick of timber. Or as the homely English proverb expresses it: "you can not make a silk purse out of a sow's ear."

But we must hasten to consider secondly——

II. The *Method of Study*. Method in study is invaluable to the student. Without it little can be accomplished. Few will deny this; and yet most will ask—what is the best method of study? We will here give you results principally of our own long experience.

First, then:—

1st. *Fix your attention upon the particular subject before you.* Suffer not your thoughts to wander; give your whole mind to the matter. Never be satisfied with hasty, superficial views; penetrate the full depths of your author's meaning.

Much is lost from a want of attention. A dreamy, listless mood can lead to nothing but vague, indefinite, shallow views. Such a student never arrives at certainty; and he can have little confidence in himself, neither ought he to have. No man can ever be thoroughly acquainted with any one point, much less with the entire subject, who does not concentrate his whole mind, bring all his powers to bear upon the study before him. Again:—

2d. *Do not limit yourself to one branch of study at a time.* The soundness of this rule may be seen in the very nature of the mind. After a time—sooner or later in each one—the mind becomes weary of the matter before it; it begins to flag, and lose all power of attention. Now, so soon as this sense of weariness gets the upper hand of us it is wise to stop, because after this no real advantage is gained, no substantial improvement made.

But must we then do nothing? Certainly not; for it is a law of the mind that, unless completely jaded, it will awaken afresh, and acquire new vigor, simply by turning away from what is before it, and directing its attention to another subject. Every experienced student is sensible of this.

When, then, you find your mind losing its grasp of one subject, turn to another. Lay down, for example, your Greek or Latin classic, and take up some mathematical treatise; and when this also has wearied you, turn to intellectual philosophy, or ethics, or political economy; and when here again your attention flags, go to some work in natural philosophy; and then pass on to history, poetry, literary criticism, the periodicals of the day and similar reading.

We do not mean that all these subjects must be taken up on each occasion, for neither your time nor your strength will always allow this. But let your method be to turn from one subject to another as your mind becomes wearied by any one. In the very act of turning the attention is revived, and the whole mind reinvigorated. Hence you may in this way improve your time and opportunities to the uttermost.

When, however, you find that your mind does not readily recover itself by change—this is a sure indication that you have done all that you ought to do at that time. To go further is worse than useless; you will not only make no real progress in knowledge, but you will injure your health both of body and mind. Now throw down your books, dismiss all subjects of study, and go to splitting wood, or take a long walk, and, in this case, see to it that you do some good by the way to your fellow creatures. Such physical exercise, especially when accompanied by benevolent endeavors to relieve the suffering, or in any way promote the welfare of others, will most happily tend to draw off your mind from its usual pursuits, give it the best chance of recovery and so send you back again with recruited energies, and in every way better fitted for study. Further:—

3d. *Do not waste your time in reading poor books, much less bad books.* A bad book may as effectually corrupt the heart and ruin the man as the practice of vice. Indeed, such reading is itself vicious. And even authors that are light and superficial will be sure to weaken the mind and unfit it for vigorous effort.

Get not only good books, but the very best upon the subject; one such will be of more benefit to you than a score of inferior volumes. Again:—

4th. *Read always with pen or pencil in hand.* Make notes upon every book you study. Do this upon the fly leaves; and if these are not sufficient, fasten more blank paper in the back or front of the volume.

If your author mentions a valuable fact or incident, make a reference to the page; if a very important or original idea, mark its place; or if you meet with some peculiarly felicitous expression, or some passage of remarkable vigor or beauty of style, note it for future study; if you find any incorrect statement, or illogical argument, or error, be sure, at the time, to write as full and clear a refutation of it as possible.

By this careful, diligent reading, you can not fail to rise up fully acquainted with your author, and, not unfrequently too, with a more correct and thorough knowledge of the subject than the author himself had. You will thus become "wiser than your teachers;" be sure, however, to cherish a grateful sense of your indebtedness to them for having put you in the path to truth and for all the help they have given you by the way.

But besides storing your mind with valuable knowledge, and almost indelibly impressing it upon your memory, this method of making notes upon authors will enable you, years afterward, by glancing at your references, to recall all that is valuable in a book, and give you a ready access to its contents.

Another advantage of this method of making notes,—you will gradually accumulate, if your studies have been widely and diligently pursued, such a fund of materials in the various departments of science and literature, and such a facility of reference to authorities, as will best prepare you for lecturing, or for authorship yourself, or for contributing to the periodicals of the day, essays, reviews, and digests of authors and subjects. No one who has faithfully persued the method of study here indicated can fail to discover its value in this respect.

Some are in the habit of keeping common place books, and, I doubt not, they find them very useful. But I have never done this. I have rather trusted to my memory thus thoroughly exercised by close study and writing notes upon each author. And as the result—I here state it for your encouragement—frequently in writing discourses, addresses, or treatises upon particular subjects, I can readily recall important principles, views, trains of thought, and facts, or passages of striking force or beauty; and remember in what authors these occur, and turn at once to the places they occupy, or speedily find them by my notes on the fly-leaves, though twenty years or more may have elapsed since I

studied the author, and I scarcely ever opened the volume since.

Every one will readily perceive that the acquisition of such a power is invaluable to the student, whether he be a professional man, or a public man, an author, an editor, or filling a professor's chair. Furthermore :—

5th. *Be careful to treasure up your odd moments.* When, perhaps, having finished some professional or other engagement, you found yourself at leisure—have you not at times thus said to yourself: "The dinner bell will ring in a moment; or Mr. So and So will be here in a moment, as he promised; or it is only a moment before I must go and fulfill such and such an appointment; I have no time therefore to take up a book."

But have you not often found that the dinner bell did not ring as soon as you thought it would? or, that the individual you were waiting for, did not meet his appointment as he promised? or, that, for some good reason or other, you delayed to go forth as you intended? And so you sat listlessly, or still worse, impatiently waiting; and in this way lost both your time and your temper. Whereas, had you taken up a volume, you might have been most profitably and happily employed. Any one who will try this plan of putting his odd moments to the best account in study, will be amazed, when he looks back upon the precious fragments he has thus gathered up.

Do not then risk your time or your temper upon the punctuality of others, or upon the uncertain events of life. Treasure up these odd moments; they will be sure to render you a wiser and a better man.

Let me here exemplify these remarks by a single case. Doubtless you have all heard of Dr. John Mason Good, the distinguished physician of London. No man was ever more diligent in business, and at the same time more devoted to study. Not a moment would he permit to pass away unimproved. Take one instance of his wise and careful use of time. At a certain period of his life, he was accustomed, before leaving home, to visit his patients, to tear a few pages from the Latin classic poem of Lucretius—"De Rerum Natura;" and as he was passing along the streets, instead of wasting his time in gazing upon the signs, or passing persons or events, he would, by a few glances at the pages he had brought with him, fix these in his memory, and then turn them in his mind into English verse, for which he had acquired a wonderful facility. When he returned home, after entering an account of his professional services, he would take out of his pocket the leaves of Lucretius, the bare sight of which would recall his poetical version. This he would then put upon paper. So he persevered till he finished the whole; and then he added to it a vast body of notes containing an invaluable amount of classical, sacred, and oriental learning. The work was published in two volumes, *quarto;* and had Dr. Good never written any thing else, this work alone would have deservedly placed him in the front rank of scholars. But it was only the fruits of his odd moments; and it is only one of the numerous and able productions with which this truly wise and diligent man enriched the cause of learning, and adorned his profession. Again:—

6th. *Compose much, and speak much.* Suppose yourselves, my young friends, standing in the garret of a house, and looking round, you saw here a rich center table, there a splendid pier glass, in this place a magnificent sofa and beautiful ottomans, in that a superb side-board with highly wrought chairs of various size and patterns, together with costly carpets, rugs, rich china wares for every use, chandeliers, tasty curtains, and kitchen furniture of every sort: and all these scattered in confusion, or piled up indiscriminately in huge heaps. "Of what use," you would exclaim, "of what use can this costly furniture be? It might as well be sunk into the depths of the ocean, or burned up, or buried in the bowels of the earth, for any good it does here to its owner or others."

Suppose again, my young friends, you walk through a spacious dwelling, and see it abundantly and richly furnished from bottom to top, and every thing in its place,—the parlors, reception rooms, sitting rooms, chambers, closets, kitchen—all admirably arranged for convenience and comfort; and good taste also consulted throughont. "Here," you would say, "is wisdom; with what judgment has every thing been disposed There is nothing wanting for use or enjoyment. Such a home is a treasure; who would not wish to possess it? Every guest is tempted to repeat his visit. All is convenient, elegant, and fit for its right use."

Now, my young friends, is not such an apt representation of the merely learned man, and of the thoroughly educated man? The knowledge of the first, vast and multifarious as it may be, is like furniture piled away in a garret, of no use to its possessor or any body else. But the thoroughly educated man has his rich acquisitions all suitably arranged—every thing in its place. The taste is delighted, as well as every want met. And he can put all he has to immediate and most efficient use for himself and others. Hence the well educated man will be sure to command respect. Society will count him among its most valuable members.

But some may here ask—how can I be, not merely a learned man, but a thoroughly educated man? We answer—besides applying your mind most faithfully for the acquisition of knowledge, you must accustom yourself daily to write and to speak upon whatever has occupied your mind, and, so far as you can, upon what others, who have a right to your efforts, may desire to be instructed in by you.

By attempting to write upon a subject you will soonest discover how very imperfect is your knowledge of it; this will humble you, and lead you to more diligent, careful, and thorough research. By writing much your knowledge will also become well methodized—every thing will find its proper position; and you will acquire a continually increasing facility of making all you really know at once available for your own use and that of others. The same holds true of speaking also, though not to so great an extent.

Of what possible benefit are a man's largest intellectual acquisitions if these are not well arranged, and he can not give them ready utterance? They are just like so much furniture heaped away in a garret. I would rather know but little, and that little well, and be capable, on every suitable occasion, of bringing it out with clearness and interest by my pen or my tongue. I should feel in this case that I was of some use in the world, and that I deserved to be a respected and cherished member of the community. We may here add another remark. It is only the professional man who can write or speak with ease that is likely to advance the learning and honor of his profession. Once more:—

7th. *Prayer.* Besides the many commands and promises of God to us all in respect to prayer—besides these, is there not that in the very constitution of the mind itself which must ever render prayer of peculiar importance to the student? He who would use all the powers and resources of his mind to the best advantage, must be able at all times to have these completely at his command. But such readiness and self-control imply a calm spirit and great energy of will. Now, nothing tends so much to compose the mind and still every agitation of feeling, and, at the same time, give determination to the will, as prayer. He who sincerely prays, must have more or less confidence that he has God Almighty on his side; and consequently that he has nothing to fear, and that he can do all things fit for him to do,—all things really profitable to himself and others, and to which duty calls him.

Hence, I have always observed that pious young men, other circumstances being equal, were generally the best students and stood highest in their classes: and that even those who merely respected religion and paid more or less attention to its observances, though not really pious, were better students than the thoughtless and decidedly worldly. A grossly immoral man, I need scarcely add, can rarely excel in any branch of learning that requires close application. Such will have neither the disposition, nor that elevation of aim, nor that strength of purpose which are needful to perseverance in study.

But the importance of prayer to the student is too extensive a theme for a single head of a discourse. We will therefore close our remarks by simply pointing you to the example of Lord Bacon, the father of modern philosphy. That great man, with all his intellectual powers and vast stores of learning, still felt his utter insufficiency as a student and an author without God's help and blessing. Hence you will find in his works some specimens of his prayers—equally remarkable for their child-like simplicity of spirit and sublimity of thought.

Listen to an extract from one of his prayers as a student: "Illuminate the eyes of our minds and understandings with the bright beams of thy Holy Spirit, that we may daily grow in the saving knowledge of the heavenly mysteries of our redemption wrought by our dear Lord and Saviour, Jesus Christ; sanctify our wills and affections by the same Spirit, the most sacred fountain of all grace and goodness; reduce them to the obedience of thy most holy will in the practice of all piety towards thee, and charity towards all men!" Hear him, again, as a student thus pouring out his heart to the Almighty:—"We humbly and earnestly beg, that human things may not prejudice such as are divine; neither that from the unlocking of the gates of sense, and the kindling of a greater natural light, any thing of incredulity or intellectual night may arise in our minds towards divine mysteries; but, rather, that by our mind thoroughly cleansed and purged from fancy and vanities, and yet subject and perfectly given up to divine oracles, there may be given unto faith the things that are faith's!" But, once more,—hear how he prays, as an author, before publishing a production of his pen—"Thou, O Father, who givest the visible light as the first born of thy creatures, and didst pour into man the intellectual light as the top and consummation of thy workmanship, be pleased to protect and govern this work, which, coming from thy goodness, returneth to thy glory!" We now proceed, thirdly and finally to—

III. *The End of Study.* That we may clearly see what ought to be the great *end* of study, let us suppose that you have possessed all the advantages and opportunities of which we have spoken; and that you have duly improved these.

If it be thus with you, surely some such thoughts as these are very natural and highly suitable to you—so much so that we see not how they could fail to occur to you:—"I find myself, by God's gift, in the possession of a good share of mind; I have had, under providence, liberal means of improvement afforded me; I have been enabled diligently to make use of these; so that I now have acquired such knowledge as, if properly employed, is capable of making me very useful and highly respected. Surely I ought to make a suitable return to the Giver of all good, and I ought to be very happy in the lot he has seen fit to assign me. But, alas! I find that hitherto I have thought too little of God, too little of my own highest interests. I have lived for self and the world. I am verily guilty in the sight of heaven; and not only so, but I find in myself so much of weakness and proneness to evil, that in spite of all my good resolutions, I have not as yet sought first the kingdom of God and his righteousness.

"How then shall I obtain pardon for the past, and find wisdom and strength for the future to form and pursue such plans of life as will please God, satisfy my own conscience, advance me most in every true excellence, and exert the best possible influence over others?—so that when called hence I shall find it my happy privilege to go into eternity with a good hope through grace of never ending blessedness; and with a well grounded confidence also that I leave the world the wiser and better for my example in it? Should I fail in these things, whatever else I may gain, of learning, or wealth, or worldly honor,—I have failed in the great purpose of life, I shall make shipwreck of myself for time and eternity.

"How then may I obtain these precious benefits?—how be safe, useful, happy here, and prepared for the higher usefulness and blessedness of eternal life?"

Are these, my young friends, *your* thoughts—these the deep ponderings of *your hearts?* Do you wish light and satisfaction here? We thank God, if it is so with any of you; for such reflections and desires are the strivings of his Spirit in your bosoms. Do not resist them, we beseech you. Yield to them now,—they will lead you onward and upward, onward and upward to that good part which shall not be taken away from you.

Go then, in this teachable, enquiring, serious frame,—go to your Bible—God's message of grace and mercy to a lost world. And while you will find God's book dealing honestly with you,—showing in the light of his holy, unchangeable law, your guilt and ruin through sin; you will yet discover in it all your heart can wish in its deepest needs and largest desires. In that simple, but stupendous scheme of redeeming love brought to light by the gospel, you will see how the Holy Sovereign of heaven and earth can hold out the golden sceptre of mercy to the chief of sinners, and yet uphold the pillars of his righteous government in all their strength and grandeur. Sitting by faith at the feet of Jesus, clothed in his righteousness and renewed in the spirit of your mind, it will be your happy privilege to have the peace of God in your conscience, and his love shed abroad in your heart by the Holy Ghost given unto you.

Your views, your feelings, your wishes, your hopes, your aspirations will thus all become *new*,—so wonderful, excellent, soul-satisfying, NEW,—that you will then see you never knew before what true happiness was; your heart will melt in gratitude, and your mouth be filled with thanksgiving and praise.

And thus joyfully receiving the truth and spirit of Jesus—

your God and Savior—you will find in yourself a clearness, and a vigor of mind, and an energy of purpose to which you were before a stranger. Hence you will come to all your studies with a warmer, a nobler zeal to serve God in them, and to be increasingly useful to your fellow men—*the true end of study!*

Such a spirit will raise you above the petty jealousies and vexations of the world, while it will call forth into lively exercise every thing within you and around you, calculated to render you daily a wiser, a better and a happier man; ensure you increasing respect and success in your profession; and sustain you at every step in life with the growing assurance of that higher scene of usefulness, excellence, and blessedness which awaits you in God's eternal kingdom.

